# Antimicrobial susceptibility profiles of commensal *Clostridium perfringens* isolates from chickens in Hungarian poultry farms between 2022 and 2023

**DOI:** 10.3389/fvets.2025.1589747

**Published:** 2025-07-25

**Authors:** Ádám Kerek, Ábel Szabó, Franciska Barnácz, Bence Csirmaz, László Kovács, Ákos Jerzsele

**Affiliations:** ^1^Department of Pharmacology and Toxicology, University of Veterinary Medicine, Budapest, Hungary; ^2^National Laboratory of Infectious Animal Diseases, Antimicrobial Resistance, Veterinary Public Health and Food Chain Safety, University of Veterinary Medicine, Budapest, Hungary; ^3^Department of Animal Hygiene, Herd Health and Mobile Clinic, University of Veterinary Medicine, Budapest, Hungary; ^4^Poultry-Care Kft., Újszász, Hungary

**Keywords:** *Clostridium perfringens*, antimicrobial resistance, minimum inhibitory concentration (MIC), PCR, chickens, Hungary

## Abstract

**Introduction:**

One of the most pressing challenges of our time is the global spread of antimicrobial resistance (AMR). Regular surveillance studies are critical for advancing collaborative efforts between animal and public health sectors, aligning with the One Health concept.

**Methods:**

In this study, we aimed to assess the antibiotic susceptibility of commensal *Clostridium perfringens* (*C. perfringens*) strains (*n* = 146) isolated from largescale poultry farms in Hungary, using minimum inhibitory concentration (MIC) determinations. Additionally, PCR was employed to investigate the presence of major and minor virulence factors.

**Results:**

Our findings revealed a decrease in the efficacy of penicillin-based antibiotics, which are primary choices for treating *C. perfringens*-related infections, with resistance observed in 48.3% of isolates for penicillin and 20.7% for amoxicillin. Furthermore, virulence gene analysis identified 47 strains (32.2%) carrying the major *beta* toxin gene, one strain with the *epsilon* toxin gene (0.7%), 27 strains (18.5%) with the minor *beta2* toxin gene, and four strains (2.7%) with the *netB* toxin gene.

**Discussion:**

These results underscore the necessity of regular surveillance studies and highlight the significant role of commensal strains as reservoirs for sustaining resistance. Future research should include larger sample sizes to provide a more comprehensive understanding of resistance dynamics. Additionally, the genetic basis of resistance in multidrug-resistant strains should be elucidated using next-generation sequencing, enabling targeted interventions to address this growing concern.

## 1 Introduction

Bacteria can develop resistance to antimicrobial agents which previously were effective in treating infections caused by them. This phenomenon, known as antimicrobial resistance (AMR), represents a global challenge (1). Various microorganisms, such as fungi and saprophytic bacteria, have evolved mechanisms to produce antimicrobial compounds as part of their natural defense strategies (2). The production of antibiotic compounds provides an evolutionary advantage by inhibiting competitors in the struggle for resources (3). Additionally, antibiotics may function as signaling molecules in intercellular communication under natural conditions (4). While many antimicrobial agents are derived from natural compounds, modified to enhance their efficacy, others, such as sulfonamides and fluoroquinolones, are fully synthetic (2). The spread of AMR has emerged as one of the most pressing health concerns in both veterinary and human medicine (1). Consequently, extensive research efforts now focus on exploring alternatives to replace antibiotics partially or entirely. These alternatives include plant-based essential oils (5), plant extracts (6, 7), antimicrobial peptides (8), and propolis, which has demonstrated antimicrobial activity in multiple studies (9–11). Furthermore, medium-chain fatty acids and triglycerides also possess antimicrobial properties (12). The poultry industry, following the swine sector (13) ranks as the second-largest consumer of antibiotics in animal agriculture, underscoring the critical need for responsible and regulated use of antimicrobials within the sector (14). Effective biosecurity measures further play a vital role in reducing antibiotic use (15). To preserve the efficacy of antimicrobial agents, susceptibility testing, coupled with pharmacokinetic and pharmacodynamic studies, is essential before initiating therapeutic treatments (16).

*Clostridium perfringens* (*C. perfringens*) is a Gram-positive, anaerobic, spore-forming bacterium most commonly associated with necrotic enteritis in poultry, particularly broiler chickens, where it has significant economic consequences (17). Subclinical infections alone are estimated to cause losses of up to five cents per bird (18), while outbreaks globally result in a $2 billion annual loss to the broiler industry (18). A recently developed toxin-based classification system categorizes *C. perfringens* strains into seven types (A–G) based on their toxin production, which determines their pathogenicity (19). These types are associated with various diseases: for example, enterotoxin-producing strains (CPE) are classified as type F, while strains producing the critically important virulence factor *netB* toxin are grouped under type G, encompassing isolates responsible for necrotic enteritis (19). The presence of AMR and virulence factors can be interdependent, with antibiotic selection pressure exerting a positive or negative correlation with these traits, potentially influencing the pathogenicity of the microorganism (20–22). As an opportunistic pathogen, *C. perfringens* is part of the microbiota in the gastrointestinal tracts of healthy humans and animals but can also contribute to various diseases (23). It is a frequent cause of foodborne illnesses, typically linked to the consumption of fresh vegetables and fruits contaminated with *C. perfringens*-bearing soil or water, as well as improperly handled meats and cooked foods. The heat-resistant spores of the bacterium can germinate and proliferate rapidly in cooled foods. Additionally, seemingly healthy animals can transmit *C. perfringens* to humans through direct or indirect contact, potentially causing disease in new hosts (24).

In the treatment of anaerobic bacteria such as *C. perfringens*, penicillin remains a viable option (25). Amoxicillin, belonging to the class of time-dependent bactericidal antibiotics (26), is effective not only against Gram-negative infections but also in treating *C. perfringens* (27). Optimal dosing aims to ensure that the drug concentration exceeds the minimum inhibitory concentration (MIC) for 40–60% of the dosing interval, thereby achieving the necessary T > MIC threshold (28, 29). Most *Clostridium* species are non-pathogenic, with only a few identified as disease-causing. Their versatile metabolism allows them to degrade a wide variety of organic materials through multiple metabolic pathways, including proteins, peptides, amino acids, sugars, purines, pyrimidines, alcohols, and organic acids (30). This versatility provides them with an essential ecological role in biomass renewal (31). However, the historical overuse of antibiotics has contributed to the spread of AMR in *Clostridium* strains and recent studies have shown a decline in the sensitivity of *C. perfringens* isolates from food samples to tetracycline, erythromycin, and lincomycin (32).

Monitoring trends such as these is crucial for tracking AMR, which is crucial to combat and it thus underscores the importance of our study. We aimed to assess the antimicrobial susceptibility profiles of commensal *C. perfringens* strains isolated from large-scale poultry farms in Hungary. Additionally, we explored potential correlations between the presence of virulence factors, identified through PCR assays, and the resistance profiles of these strains.

## 2 Results

### 2.1 Regional distribution and origin of samples received

**Figure 1:**
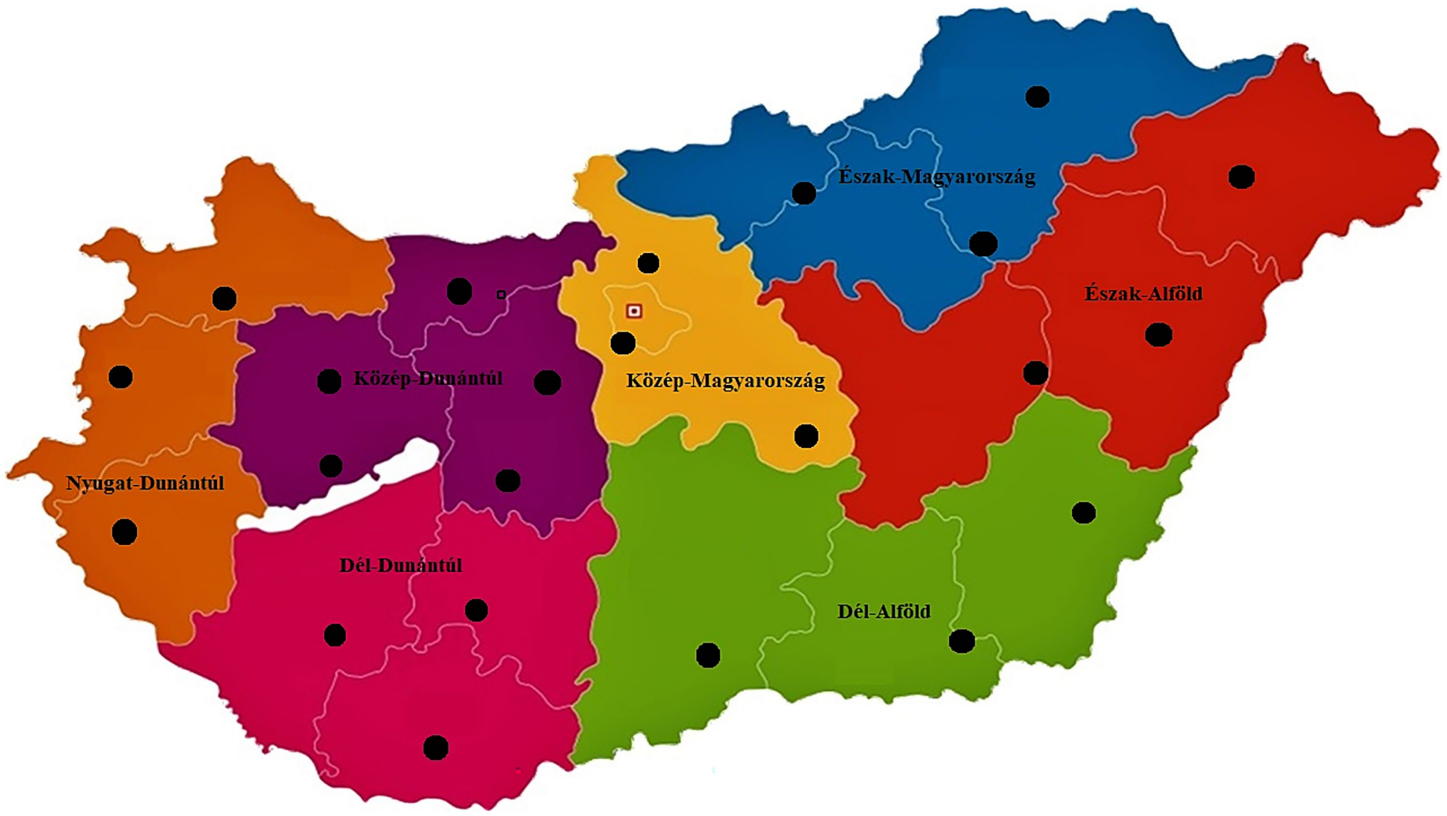
Regional origin of samples collected from chickens across the seven regions of Hungary. Black dots indicate the geographic locations (*n* = 23) of sample sources, demonstrating nationwide coverage.

We examined a total of 146 *C. perfringens* isolates obtained from broiler chickens. The regional distribution of the isolates (Figure 1) was as follows: 8 isolates (5.5%) originated from the Dél-Alföld region, 23 isolates (15.8%) from the Dél-Dunántúl region, 24 isolates (16.4%) from the Észak-Alföld region, 17 isolates (11.6%) from the Észak-Magyarország region, 32 isolates (21.9%) from the Közép-Dunántúl region, 14 isolates (9.6%) from the Közép-Magyarország region, and 28 isolates (19.2%) from the Nyugat-Dunántúl region. These seven regions correspond to all NUTS-2 statistical regions of Hungary, providing nationwide geographic coverage in the isolate sampling.

Among the isolates, 39.7% were derived from broiler flocks, 35.6% from breeding flocks, and 24.7% from egg-laying flocks. Additionally, 39.7% of the isolates originated from juvenile flocks, while 60.3% were from adult flocks. Regarding flock size, 42.7% of the isolates were obtained from small flocks (5,001–50,000), 35.6% from medium-sized flocks (50,001–100,000), and 21.7% from large flocks (>100,001).

### 2.2 Correlation and clustering of resistance profiles

**Figure 2:**
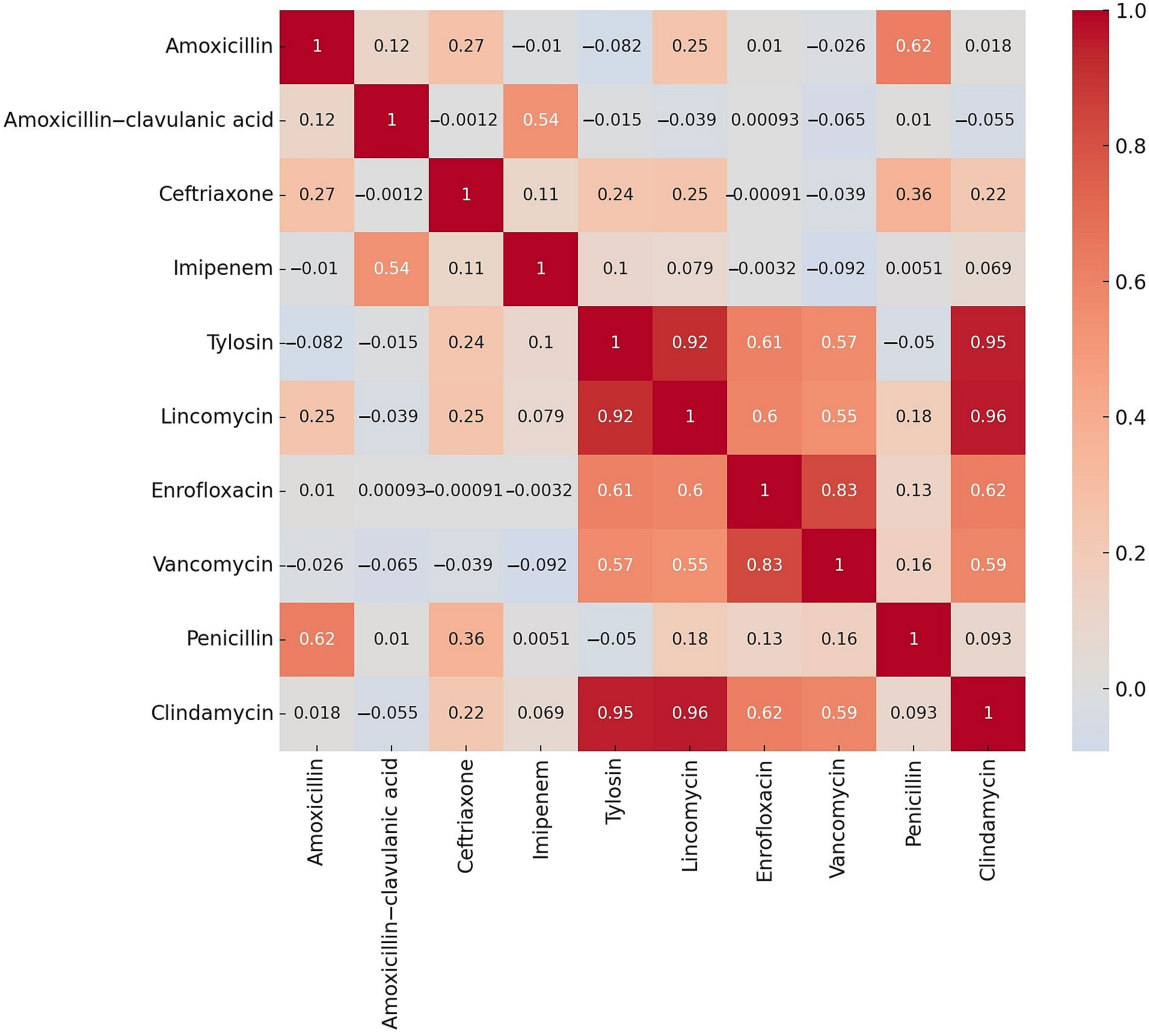
Correlation analysis of resistance rates between various active substances following the determination of resistance levels in *Clostridium perfringens* strains (*n* = 146) isolated from domestic chickens.

After determining the MIC values, we assessed the resistance rates for antibiotics with established clinical breakpoints. Subsequently, correlation analysis was performed to evaluate the relationships between the different active substances (Figure 2). We observed a strong positive correlation between lincomycin and clindamycin (0.96), tilozin and clindamycin (0.95), tilozin and lincomycin (0.92), as well as enrofloxacin and vancomycin (0.83).

**Figure 3:**
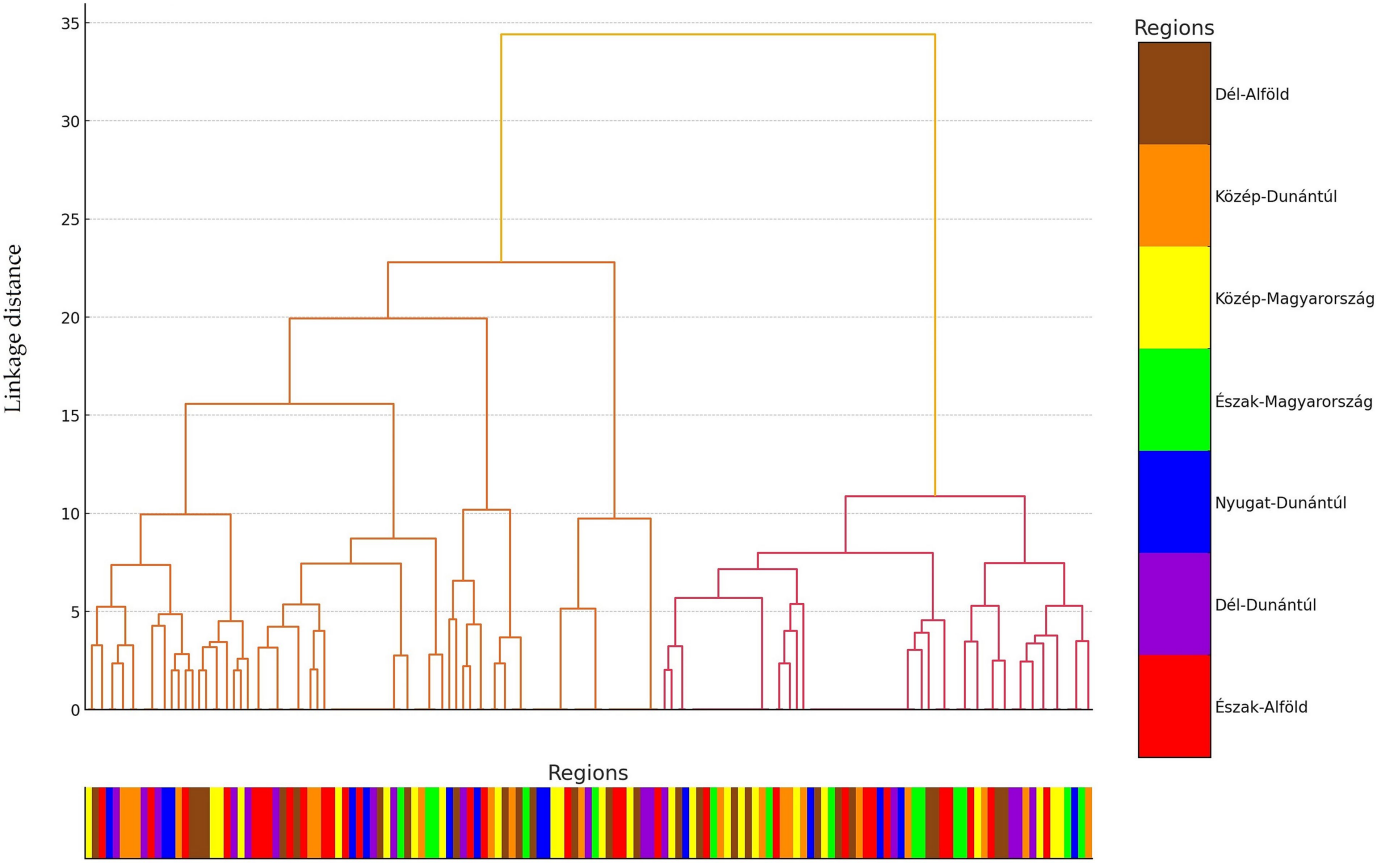
Hierarchical cluster analysis of commensal *Clostridium perfringens* strains (*n* = 146) isolated from chickens. The branches of the dendrogram represent clusters of isolates with similar resistance profiles, while the branch lengths indicate the degree of similarity or difference among the isolates.

**Figure 4:**
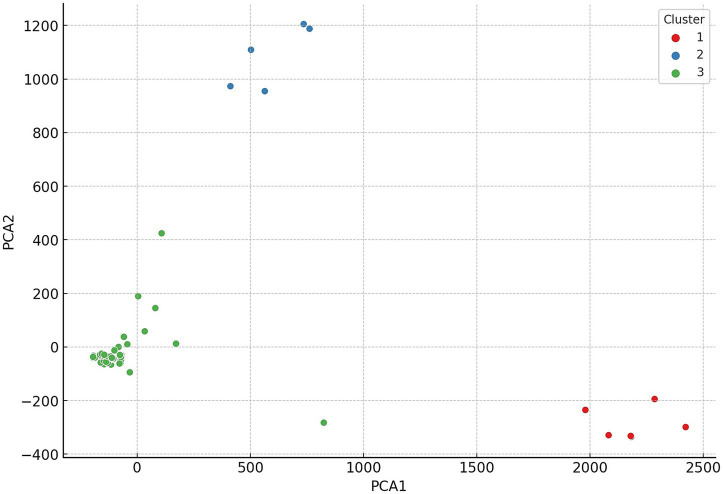
Principal component analysis (PCA) of commensal *Clostridium perfringens* strains (*n* = 146) isolated from chickens. The first principal component accounted for the largest variance, followed by the second principal component. Only a few strains were observed in the third cluster.

Hierarchical cluster analysis was conducted to uncover patterns and relationships among the examined strains based on their antimicrobial resistance profiles. Based on the clustering results, the 146 commensal *C. perfringens* strains were grouped into three main clusters, which reflect distinct resistance profiles. These hierarchical relationships are visualized in a dendrogram (Figure 3), which highlights structural similarities and potential outliers. The three clusters are further illustrated in the principal component analysis (PCA) plot (Figure 4), where they are color-coded for clarity.

Subsequently, PCA was performed to reduce dimensionality and uncover major patterns in the antimicrobial resistance profiles (Figure 4). The first principal component accounted for the largest proportion of variance, while the second component, orthogonal to the first, explained the next highest amount. This analysis revealed clear clustering tendencies among the strains. The geographic origin of isolates strongly correlated with cluster membership: strains from the same region were predominantly grouped within the same cluster, a pattern supported by the horizontal color-coded axis. The cluster analysis revealed significant associations between certain antimicrobial resistance profiles and cluster membership. For instance, isolates resistant to lincosamides (lincomycin and clindamycin) were predominantly assigned to the second cluster. Additionally, the results suggest geographic patterns likely influenced by region-specific antibiotic usage practices.

### 2.3 Regional and usage-related patterns of resistance

Statistical analyses were conducted to evaluate resistance levels for each active substance based on utilization type (Table 1). Significant differences in resistance were observed between egg-laying and meat-producing flocks, as well as between breeding and meat-producing flocks, particularly for lincomycin, enrofloxacin, penicillin, and clindamycin. In all cases, broiler chickens exhibited significantly higher resistance levels compared to other utilization types.

**Table 1:** Importance of each type of utilization in the resistance profile, by antibiotics.

Antibiotics	Laying–breeding	Laying–broiler	Breeding–broiler
Amoxicillin	0.3372	0.0674	0.3075
Imipenem	0.2455	0.1751	0.7416
Lincomycin	0.7520	0.0002*	<0.0001*
Enrofloxacin	0.6347	<0.0001*	<0.0001*
Penicillin	0.0929	0.0004*	<0.0001*
Clindamycin	0.1367	0.0210*	0.0170*

*significant difference (*p* < 0.05).

The influence of age groups on resistance levels was also assessed for each active substance (Table 2). Significant differences were observed for lincomycin, enrofloxacin, penicillin, and clindamycin, with notably higher resistance levels identified in juvenile flocks compared to adult populations.

**Table 2:** The importance of age group on the degree of resistance to each antibiotic.

Antibiotics	^1^Young–^2^adult
Amoxicillin	0.0954
Imipenem	0.3563
Lincomycin	<0.0001*
Enrofloxacin	<0.0001*
Penicillin	<0.0001*
Clindamycin	<0.0001*

*significant difference (*p* < 0.05).

1 Younger than 6 weeks.

2 Older than 6 weeks.

Additionally, farm size was evaluated as a potential factor influencing resistance levels across the tested active substances (Table 3). The most notable differences were observed between small and large-scale farms for imipenem, lincomycin, and enrofloxacin. In all cases, significantly higher resistance levels were identified on large-scale farms compared to smaller ones.

**Table 3:** The effect of colony size on the level of resistance to certain antibiotics.

Antibiotics	Small–medium	Small–large	Medium–large
Amoxicillin	0.6626	0.9160	0.6436
Imipenem	0.0580	0.0491*	0.9290
Lincomycin	0.3499	0.0088*	0.0521
Enrofloxacin	0.0419*	0.0043*	0.2270
Penicillin	0.1836	0.0669	0.5110
Clindamycin	0.0260*	0.0984	0.7839

*significant difference (*p* < 0.05); small (5001–50,000); medium (50,001–100,000); large (>100,001).

### 2.4 MIC distributions and susceptibility classification

We generated frequency tables for the determined MIC values for each active substance which has clinical breakpoints, including the percentage distribution of these values (Table 4). Subsequently, we calculated the MIC_50_ and MIC_90_ values and included the epidemiological cut-off values (ECOFF) defined by European Committee on Antimicrobial Susceptibility Testing (EUCAST) for clindamycin. However, ECOFF values were not the other antibiotics we tested. Both the calculated MIC_50_ and MIC_90_ values for imipenem were below the clinical breakpoint. Additionally, the MIC_50_ value was below the clinical breakpoint for penicillin, clindamycin, and amoxicillin. The ECOFF value for clindamycin was also below the clinical breakpoint. A frequency table for active substances without clinical breakpoints is provided in Supplementary Table S1.

**Table 4:** Frequency table of minimum inhibitory concentration (MIC) values for antimicrobials with defined breakpoints in *Clostridium perfringens* isolates from domestic chickens (*n* = 145).

Antibiotic	^1^BP*	0.001	0.002	0.004	0.008	0.016	0.03	0.06	0.125	0.25	0.5	1	2	4	8	16	32	64	128	256	512	1,024	MIC_50_	MIC_90_	^2^ECOFF
	μg/mL																						μg/mL		
Lincomycin	1				7	0	0	0	13	2	3	15	15	3	4	9	3	52	1	11	1	6	32	256	-
					4.8%	0.0%	0.0%	0.0%	9.0%	1.4%	2.1%	10.3%	10.3%	2.1%	2.8%	6.2%	2.1%	35.9%	0.7%	7.6%	0.7%	4.1%			
Penicillin	1		2	0	0	9	10	16	24	9	5	12	9	12	6	1	22	0	0	2	1	5	0.5	32	-
			1.4%	0.0%	0.0%	6.2%	6.9%	11.0%	16.6%	6.2%	3.4%	8.3%	6.2%	8.3%	4.1%	0.7%	15.2%	0.0%	0.0%	1.4%	0.7%	3.4%			
Enrofloxacin	2		1	0	0	0	3	2	25	14	13	3	8	10	16	43	0	0	6	1			4	16	-
			0.7%	0.0%	0.0%	0.0%	2.1%	1.4%	17.2%	9.7%	9.0%	2.1%	5.5%	6.9%	11.0%	29.7%	0.0%	0.0%	4.1%	0.7%					
Clindamycin	8				4	9	6	18	16	5	11	6	9	2	7	3	37	2	2	2	0	6	1	32	0.125
					2.8%	6.2%	4.1%	12.4%	11.0%	3.4%	7.6%	4.1%	6.2%	1.4%	4.8%	2.1%	25.5%	1.4%	1.4%	1.4%	0.0%	4.1%			
Amoxicillin	16			4	10	13	2	19	13	10	31	9	3	1	0	0	19	0	0	11			0.5	32	-
				2.8%	6.9%	9.0%	1.4%	13.1%	9.0%	6.9%	21.4%	6.2%	2.1%	0.7%	0.0%	0.0%	13.1%	0.0%	0.0%	7.6%					
Imipenem	16	1	1	2	2	16	26	14	17	8	7	3	10	10	23	5							0.125	8	-
		0.7%	0.7%	1.4%	1.4%	11.0%	17.9%	9.7%	11.7%	5.5%	4.8%	2.1%	6.9%	6.9%	15.9%	3.4%									

The table presents the frequency of MIC values for each antimicrobial agent with defined clinical breakpoints. The upper row for each antimicrobial shows the count of isolates, while the lower row displays the corresponding percentage distribution. *BP, breakpoint.

1 Clinical Laboratory Standard Institute (CLSI).

2 Epidemiological cut-off value (EUCAST).

**Figure 5:**
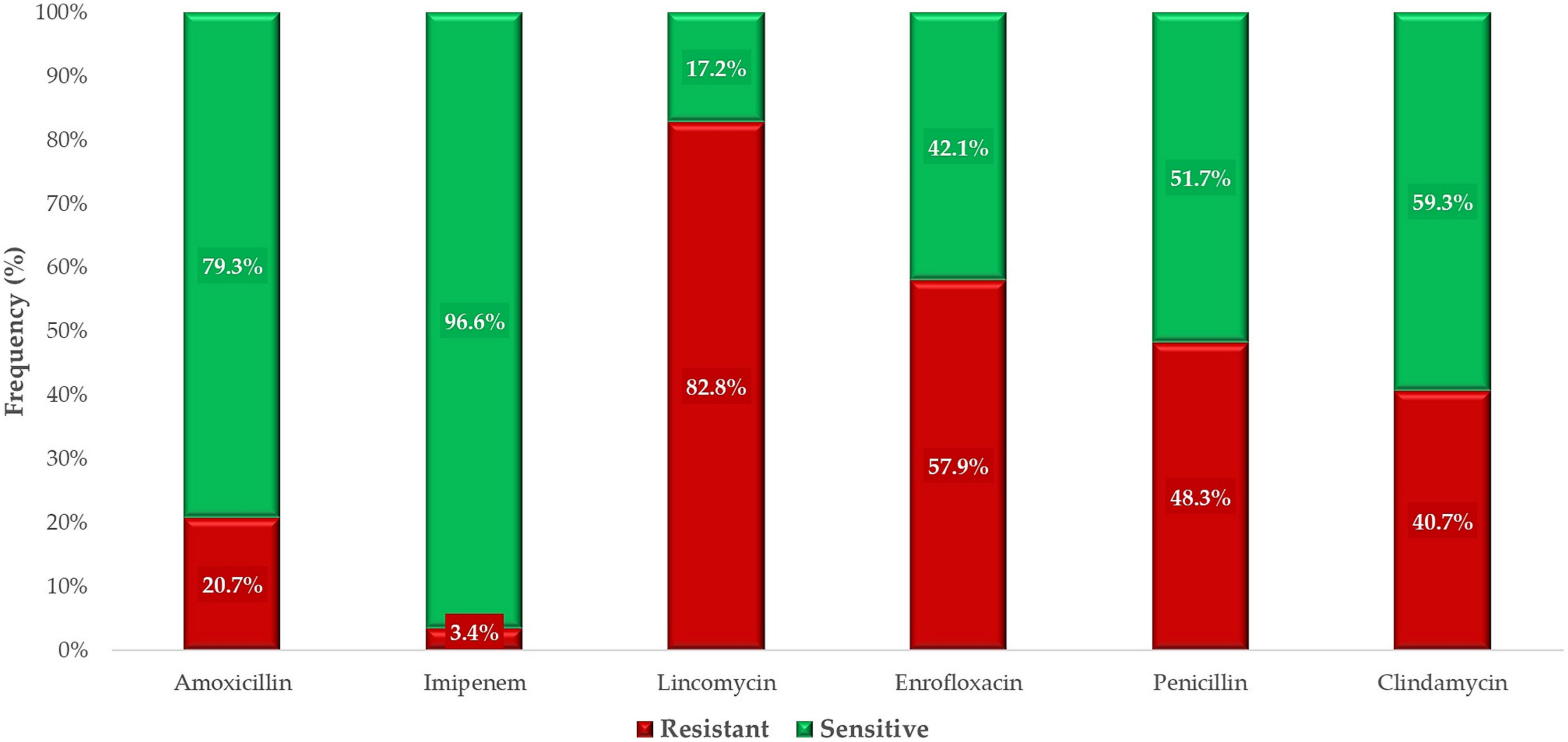
The susceptibility profile of commensal *Clostridium perfringens* strains (*n* = 146) isolated from domestic chickens to antibiotics with defined clinical breakpoints.

For antimicrobials with defined clinical breakpoints, the proportion of resistant and susceptible isolates was expressed in percentages (Figure 5). Resistance varied widely according to antibiotic, with isolates showing very low resistance to imipenem. Reduced susceptibility was observed for penicillin, with only 51.7% of isolates classified as sensitive. In contrast, 79.3% of isolates were sensitive to amoxicillin. Lincomycin was found to be the least effective, with just 17.2% of isolates displaying susceptibility.

### 2.5 Detection and co-occurrence patterns of toxin genes

We analyzed the presence of major and minor toxin genes in each isolation using PCR. All examined isolates tested positive for the major alpha toxin gene, serving as a quality control measure, as this gene is inherently carried by all *C. perfringens* strains. The major *beta* toxin gene was detected in 47 isolates (32.2%), while the *epsilon* toxin gene was identified in a single isolate (0.7%). The minor *beta2* toxin gene was present in 27 isolates (18.5%), and the *netB* toxin gene was found in four isolates (2.7%). None of the isolates carried the genes responsible for *entero* or *iota* toxin production.

When analyzing the co-occurrence of multiple toxin genes (excluding the universally present major *alpha* toxin gene), the most frequent combination—observed in 18 cases (12.3%)—was the major *beta* and minor *beta2* toxin genes. This was followed by the concurrent presence of the major *beta*, minor *beta2*, and *netB* toxin genes in seven cases (4.8%), and the major *beta*, minor *beta2*, and *epsilon* genes in three cases (2.1%). Additionally, the combination of major *beta* and *epsilon* toxin genes and that of major *beta* and *netB* toxin genes were each observed in a single instance (0.7%).

### 2.6 Association of toxin genes with resistance

Statistical analysis revealed that the presence or absence of specific toxin genes influenced the degree of resistance to certain antibiotics. For enrofloxacin, a significant difference (*p* = 0.0049) was noted when the *netB* gene was present, with significantly lower resistance levels observed in isolates carrying the gene. Furthermore, when examining the impact of carrying multiple genes simultaneously, it was found that resistance to lincomycin was significantly higher (*p* = 0.0379) in isolates lacking multiple toxin genes compared to those carrying multiple genes.

## 3 Discussion

We examined a total of 146\u00B0*C. perfringens* isolates derived from broiler chickens. Following the assessment of resistance levels, a strong positive correlation was identified between several antibiotics, including lincomycin and clindamycin (0.96), tilozin and clindamycin (0.95), tilozin and lincomycin (0.92), and enrofloxacin and vancomycin (0.83). Cluster analysis revealed that isolates resistant to both lincomycin and clindamycin were predominantly grouped within the second cluster. These findings also suggest underlying geographic patterns influencing antibiotic resistance, potentially reflecting region-specific variations in antibiotic usage.

When examining utilization patterns, significant differences in resistance levels were observed across production types, with meat-producing animals exhibiting consistently higher resistance levels compared to breeding populations for most tested substances. Furthermore, age group analysis demonstrated significantly elevated resistance to multiple antibiotics in younger, finishing animals. This trend is likely attributable to the increased selection pressure exerted by the frequent use of antibiotics during rearing. By contrast, colony size had minimal impact on resistance variability.

In *C. perfringens* infections, first-line therapeutic agents generally retained their efficacy. However, resistance patterns incorporating toxin-producing genes in the isolates revealed significant resistance differences for enrofloxacin and lincomycin, particularly when multiple toxin genes were simultaneously present.

In this study, we found that resistance to amoxicillin among the isolates was only 20.7%. This antibiotic is one of the primary choices for treating infections. However, Wei et al. (2020) reported no resistance to amoxicillin among their isolates (33). A Belgian study found that all strains isolated from the gastrointestinal tracts of broiler chickens were sensitive to amoxicillin, with MIC values below 1 μg/mL (34). In Egypt, 7% of necrotic enteritis-causing isolates were resistant to amoxicillin (35). Similarly, in the United States in 2016, no resistance to amoxicillin-clavulanate was detected in in *C. perfringens* isolates recovered from broiler chicken fecal samples (36). Comparative studies on amoxicillin-clavulanate resistance in poultry are limited. This is partly due to the lack of a maximum residue limit (MRL) for poultry, and the fact that *C. perfringens* strains typically do not produce beta-lactamase, making the addition of clavulanate unnecessary. The investigation of amoxicillin-clavulanate is therefore more relevant for research purposes than practical veterinary applications.

The reduced sensitivity to amoxicillin observed in our study, compared to other international studies, may be attributable to differing antibiotic usage practices. Oral preparations of penicillins have been extensively used in poultry production for decades, exerting significant selective pressure on the gut microbiota and contributing to environmental contamination. The 48.3% resistance to narrow-spectrum penicillin observed in this study is particularly concerning. Silva et al. (2009) reported no penicillin-resistant strains in broiler chickens, with MIC values not exceeding 0.25 μg/mL (37). Regarding imipenem, 3.4% of the isolates in our study exhibited resistance, whereas Akhi et al. reported 38% resistance in human fecal isolates (38). In Vietnam, all chicken meat-derived isolates were sensitive to imipenem (39). Although imipenem is strictly reserved for human medicine as a last-resort, life-saving antibiotic, the emergence of resistance in veterinary settings is particularly concerning and undesirable. One limitation of imipenem analysis is its instability in aqueous solutions, which may impact results (40).

We observed a resistance rate of 57.9% to enrofloxacin, while Wei et al. reported a slightly lower rate of 43.8% (33). In contrast, Osman and Elhariri documented an even higher resistance rate of 82% in isolates from broiler chickens (35). These elevated resistance rates are not surprising, given the widespread use of enrofloxacin in the poultry industry and its demonstrated ineffectiveness against anaerobic bacteria, which typically show minimal susceptibility to this drug (41). Moreover, enrofloxacin is classified as a critically important antimicrobial for human health, listed among WHO’s highest-priority fluoroquinolones (42). Its use must therefore be significantly curtailed in veterinary applications to preserve its effectiveness for treating multidrug-resistant infections in humans.

The highest level of resistance was observed against lincomycin, with 82.8% of the isolates identified as resistant. In a study conducted in Egypt, all broiler chicken isolates were found to be resistant to lincomycin (35). Similarly, two Belgian studies reported resistance rates of 61.5 and 63.3%, respectively, among isolates. However, in Brazil, 89.1% of isolates from healthy broiler chickens were found to be sensitive to lincomycin (43). For clindamycin, the resistance rate was 40.7%. In Korea, between 2010 and 2011, 52.5% of isolates from domestic chickens were resistant to clindamycin, whereas in samples collected between 2012 and 2016, the resistance rate decreased to 29.7% (33). In Canada’s Ontario province, only 2% of isolates from domestic chickens collected in the spring of 2005 were resistant to clindamycin (44). The high levels of resistance observed for both clindamycin and lincomycin can be explained by cross-resistance, as both belong to the lincosamide class of antibiotics.

The observed strong positive correlations between lincomycin and clindamycin (0.96) suggest potential co-selection mechanisms or shared resistance determinants among these compounds. Such relationships could arise from the widespread use of these antibiotics in veterinary practice, where cross-resistance is driven by overlapping resistance genes or efflux pumps targeting structurally or functionally similar antimicrobial classes. Furthermore, the overuse of lincomycin has likely exerted selective pressure which has facilitated the survival and spread of resistant strains. Inadequate dosing or suboptimal treatment durations may also have contributed to the development of antibiotic resistance, as these practices fail to eradicate bacteria completely, allowing resistant strains to proliferate. Additional contributing factors include horizontal gene transfer, genetic adaptation, and varying patterns of antibiotic use.

In the case of lincomycin and clindamycin, the nearly complete correlation reflects their classification within the lincosamide group, which shares a similar mechanism of action targeting bacterial protein synthesis. This finding aligns with previous reports indicating that resistance to one often predicts resistance to the other, driven by the *erm* genes responsible for methylation of the 23S rRNA component of the 50S ribosomal subunit. Similarly, the correlations between tilozin and both lincomycin (0.92) and clindamycin (0.95) may reflect their use in livestock production.

The correlation between enrofloxacin and vancomycin (0.83), while lower, is noteworthy and warrants further exploration. Though these antibiotics target different cellular processes, resistance may result from indirect selective pressures or the co-localization of resistance determinants on integrons or other genetic platforms. For example, quinolone resistance is most often associated with mutations in the quinolone-resistance determining regions (QRDRs) of DNA gyrase or topoisomerase IV, and it may coexist with overexpression of efflux pumps such as *norA* or *oepA* (45). Additionally, these mechanisms can occur on the same mobile genetic elements that also carry vancomycin resistance determinants (e.g., *vanA* or *vanB*), thereby enabling co-transfer of multiple resistance traits (46).

The observed differences in utilization trends are likely influenced by variations in antibiotic selection practices associated with specific production stages and age groups. Notably, adult herds may undergo multiple treatment cycles with different classes of antibiotics over their lifetime, potentially leading to a broader spectrum of selective pressures. Additionally, higher antibiotic usage in larger colonies, driven by the increased need to manage disease outbreaks and ensure productivity, may further exacerbate selection pressure, contributing to the emergence and persistence of antimicrobial resistance.

Overall, the high levels of resistance are likely the result of a combination of these factors. Implementing proper antibiotic stewardship, conducting susceptibility testing, and continuously monitoring resistance levels while investigating their genetic basis are essential measures to curb the spread of resistance.

We examined the presence of major and minor toxin genes responsible for toxin production in all 146 commensal *C. perfringens* isolates. Statistical analysis revealed that the presence or absence of specific toxin genes influenced the degree of resistance to certain antibiotics. The major alpha toxin gene was identified in every strain, consistent with findings from Lyhs et al., who also reported its presence in all isolates. However, their results were limited to isolates from animals with necrotic enteritis or those that had been euthanized (47). We identified the minor *netB* toxin gene in 2.7% of isolates. In contrast, Lyhs et al. found this gene in 8% of clinical isolates but did not detect it in commensal strains (47). The *beta2* toxin gene was detected in 18.5% of isolates, compared to only one isolate reported by Lyhs et al. (47). In contrast to the findings of Lyhs et al., who did not detect genes encoding the major beta, epsilon, or iota toxins in any of their isolates, our study identified the gene for beta toxin in 32.2% and for epsilon toxin in 0.7% of the examined isolates. Like Lyhs et al., we did not detect the major *iota* toxin gene in any strains (47). Furthermore, the minor *entero* toxin gene was not identified in any isolate. Mohiuddin et al. reported the presence of the *netB* toxin gene in two poultry isolates but did not detect genes coding for major *beta*, *epsilon*, *iota*, or minor *entero* toxins (48). In our study, statistical analysis of the presence or absence of toxin genes revealed significant findings. Notably, enrofloxacin resistance was significantly lower in our isolates harboring the *netB* gene (*p* = 0.0049), indicating a possible link between virulence and susceptibility profiles. In contrast, Slavic et al. reported that strains resistant to clindamycin and erythromycin were significantly less likely to carry the *beta2*-toxin gene; however, no significant relationships between antibiotic sensitivity and the presence of toxin genes was identified in isolates from other animal species, such as cattle, chickens, or turkeys (44). We also examined the effect of simultaneous presence of multiple toxin genes. The resistance to lincomycin was significantly higher (*p* = 0.0379) in strains lacking toxin genes than in those harboring multiple toxin genes. Wei et al. described significant differences between *C. perfringens* isolates from healthy chickens and those with clinical signs of necrotic enteritis. Specifically, isolates from diseased birds showed significantly reduced sensitivity to clindamycin compared to isolates from healthy birds (33).

Our findings underscore the critical importance of continuous monitoring of antimicrobial resistance patterns in both commensal and clinical *C. perfringens* strains. Future studies incorporating larger sample sizes, clinical isolates, and advanced molecular techniques, such as next-generation sequencing, could provide deeper insights into the relationships between resistance mechanisms and virulence factors. These efforts will be pivotal in developing effective, evidence-based antibiotic use strategies and preserving the efficacy of critically important antimicrobials for both veterinary and human health.

## 4 Materials and methods

### 4.1 The origin of the strains

The examined strains were collected between February 2022 and May 2023 during routine diagnostic investigations conducted by veterinarians serving large-scale livestock farms in collaboration with poultry health experts of the Department of Animal Hygiene, Herd Health and Mobile Clinic of University of Veterinary Medicine, Budapest. Samples, including 15 cloacal swabs per farm, were collected using Amies-type transport medium (Biolab Zrt., Budapest, Hungary). Samples were obtained from the intestinal contents or cloacal swabs of clinically healthy broiler chickens and submitted to the national reference laboratory. For the isolation of *C. perfringens*, samples were streaked onto CHROMagar™ *C. perfringens* selective agar (Chebio Fejlesztő Kft., Budapest, Hungary) and incubated at 37°C for 24–48 h under anaerobic conditions using the BD GasPak™ anaerobic system (VWR International Kft., Debrecen, Hungary). Colonies showing characteristic morphology were subcultured for purification. Species-level identification of the isolates was confirmed by MALDI-TOF mass spectrometry. The confirmed *C. perfringens* isolates were then provided to us for further analysis.

Pure cultures of the isolates were prepared on tryptic soy agar (Biolab Zrt., Budapest, Hungary) and stored at −80°C in a Microbank™ system (Pro-Lab Diagnostics, Richmond Hill, Canada).

Metadata accompanying the samples included information on the organ of origin (cloaca) and the specific location of collection. Based on the collection sites, the samples were categorized into Hungary’s seven administrative regions during recordkeeping.

### 4.2 Minimum inhibitory concentration determination

The phenotypic presence of AMR was determined by measuring MIC values following the guidelines of the Clinical Laboratory Standard Institute (CLSI) (49). Breakpoints were also defined according to CLSI guidelines (49) and compared with the ECOFF established by the EUCAST.

The isolates, stored at −80°C, were suspended in 3 mL of cation-adjusted Muller-Hinton broth (CAMHB) and incubated at 37°C for 18–24 h prior to testing. The MIC determinations were conducted using 96-well microtiter plates (VWR International, LLC., Debrecen, Hungary). All wells, except those in the first column, were filled with 90 μL of CAMHB. Stock solutions of the tested antibiotics (Merck KGaA, Darmstadt, Germany) were prepared following CLSI guidelines (49). Amoxicillin and amoxicillin-clavulanic acid were prepared in a 2:1 ratio using phosphate buffer (pH 7.2, 0.01 mol/L). Penicillin and imipenem were dissolved in phosphate buffer (pH 6, 0.1 mol/L), while ronidazole and metronidazole were prepared in dimethyl sulfoxide (DMSO). Distilled water was used for ceftriaxone, tilozin, tilmicosin, lincomycin, clindamycin, oxytetracycline, and vancomycin. Enrofloxacin was prepared in distilled water with 1 mol/L NaOH. From each 512 μg/mL working solution, 180 μL was added to the first column of the microtiter plate, followed by serial twofold dilutions across the plate. After the 10th column, 90 μL of excess solution was discarded, leaving 90 μL in each well. Bacterial suspensions adjusted to 0.5 McFarland standard using a nephelometer (ThermoFisher Scientific, Budapest, Hungary) were inoculated into the microtiter plates, starting from the 11th column and proceeding backward, with 10 μL per well (49). Evaluation was performed using the SensititreTM SWINTM automatic MIC reader (ThermoFisher Scientific, Budapest, Hungary) and VIZION system software version 3.4 (ThermoFisher Scientific, Budapest, Hungary, 2024). The quality control strain was *C. perfringens* (ATCC 13124).

### 4.3 PCR tests

Using PCR, we mapped the gene sets responsible for major and minor toxin production in each strain. DNA extraction was performed from bacterial suspensions using the Zymo Quick-DNA Fungal/Bacterial Miniprep Kit (Zymo Research, Murphy Ave., Irvine, CA, United States), following the manufacturer’s protocol. To disrupt the bacterial cells and release genomic DNA, a Qiagen TissueLyzer LT (Qiagen GmBH, Hilden, Germany) was used at 50 Hz for 5 min. The PCR assays utilized the Kylt *Clostridium perfringens* kit, specifically designed for real-time PCR detection of DNA (Kylt, Höltinghausen, Germany). This kit is optimized for detecting major (*alpha, beta, epsilon, iota*) and minor (*beta2, entero, netB*) toxin genes in bird-derived samples. All procedures adhered strictly to the manufacturer’s guidelines, with the extracted nucleic acids stored at −20°C until further use.

PCR assays were carried out using the CFX Opus Dx Real-Time PCR system (Bio-Rad Hungary Ltd., Budapest, Hungary). The data analysis was performed with the Bio-Rad CFX Maestro software version 5.3.022.1030 (Bio-Rad Hungary Ltd., Budapest, Hungary).

### 4.4 Statistical analysis

The data analysis was conducted using R version 4.1.0 (50). The normality of data distribution was tested using the Shapiro–Wilk test. For datasets that deviated from normal distribution, non-parametric statistical methods were employed. The Kruskal-Wallis test (51), was utilized to evaluate resistance levels across multiple sample groups. This method is particularly suitable for comparing medians when normality assumptions are not met. To further investigate group differences, *post hoc* pairwise comparisons were conducted using the Mann–Whitney U test (52), for non-parametric data and two-sample t-tests for normally distributed data. Bonferroni correction was applied to reduce the likelihood of Type I errors from multiple comparisons, although it is acknowledged that this approach may increase the risk of Type II errors (failing to detect true differences) (53).

Correlation analysis was performed to identify relationships among resistance profiles to various active substances. PCA (54) was employed to uncover underlying patterns in the data and to visualize similarities and differences across samples. PCA effectively reduces the dimensionality of complex datasets by transforming them into principal components, with each component representing a linear combination of the original variables. The first principal component captures the largest variance, with subsequent components accounting for progressively smaller variances. The orthogonality of these components ensures that they are uncorrelated, simplifying the interpretation of complex data relationships.

Hierarchical cluster analysis was also performed to identify clusters of isolates based on resistance profiles. The results were visualized using a dendrogram (55); a tree-like diagram illustrating the hierarchical relationships among clusters. This method groups observations into clusters that share similar characteristics, distinguishing them from other clusters. The dendrogram provides a clear visualization of the clustering process and the relative relationships between groups.

Correlation analysis further explored the strength and direction of relationships between variables. A positive correlation indicates that an increase in one variable corresponds to an increase in the other, while a negative correlation implies an inverse relationship. The correlation coefficient, ranging from −1 to +1, quantifies the strength of the relationship, with +1 representing a perfect positive correlation, −1 a perfect negative correlation, and 0 indicating no linear relationship.

## 5 Conclusion

In conclusion, our study highlights the significance of the global spread of AMR and underscores the importance of examining *C. perfringens* strains from both veterinary and public health perspectives. The resistance rates observed for penicillin and amoxicillin, alongside the detection of virulence factors, reaffirm the pivotal role of commensal-strains in maintaining resistance reservoirs. Our findings emphasize the necessity of regular monitoring, particularly in large-scale poultry operations, to quickly identify emerging resistance trends. Future studies with larger sample sizes and the use of next-generation sequencing to elucidate the genetic basis of resistance in multi-drug-resistant strains can provide deeper insights, ultimately contributing to the development of more effective treatment and prevention strategies.

## Data Availability

The original contributions presented in the study are included in the article/Supplementary material, further inquiries can be directed to the corresponding author.
