# Supplementary material for: Antimicrobial susceptibility profiles of commensal Clostridium perfringens isolates from chickens in Hungarian poultry farms between 2022 and 2023
Source: Front Vet Sci. 2025 Jul 25;12:1589747. doi: 10.3389/fvets.2025.1589747 (PMC12332747; doi:10.3389/fvets.2025.1589747)
Supplement: Supplementary file 1 [file Table_1.DOCX]

**Supplementary Table S1** Frequency table of the minimum inhibitory concentration (MIC) values (µg/mL) for agents without breakpoints in *Clostridium perfringens* samples derived from chickens (*n*=145). The top row for each agent shows the count, while the bottom row shows the percentage.

| **Antibiotics** | **0.001** | **0.002** | **0.004** | **0.008** | **0.016** | **0.03** | **0.06** | **0.125** | **0.25** | **0.5** | **1** | **2** | **4** | **8** | **16** | **32** | **64** | **128** | **256** | **512** | **1024** | **MIC_50_** | **MIC_90_** |
| --- | --- | --- | --- | --- | --- | --- | --- | --- | --- | --- | --- | --- | --- | --- | --- | --- | --- | --- | --- | --- | --- | --- | --- |
|  |  |  |  |  |  |  |  |  |  |  |  |  |  |  |  |  |  |  |  |  |  | **µg/mL** | |
| ***Amoxicillin-clavulanic acid** |  |  | 6 | 10 | 11 | 3 | 18 | 12 | 8 | 21 | 7 | 8 | 19 | 1 | 1 | 20 |  |  |  |  |  | 0.5 | 32 |
|  |  |  | 4.1% | 6.9% | 7.6% | 2.1% | 12.4% | 8.3% | 5.5% | 14.5% | 4.8% | 5.5% | 13.1% | 0.7% | 0.7% | 13.8% |  |  |  |  |  |  |  |
| **Ceftriaxone** | 2 | 0 | 3 | 12 | 2 | 12 | 11 | 6 | 5 | 4 | 12 | 10 | 12 | 8 | 34 | 9 | 0 | 0 | 0 | 2 | 1 | 2 | 16 |
|  | 1.4% | 0.0% | 2.1% | 8.3% | 1.4% | 8.3% | 7.6% | 4.1% | 3.4% | 2.8% | 8.3% | 6.9% | 8.3% | 5.5% | 23.4% | 6.2% | 0.0% | 0.0% | 0.0% | 1.4% | 0.7% |  |  |
| **Tilozin** |  |  | 2 | 1 | 3 | 3 | 2 | 32 | 9 | 4 | 24 | 24 | 2 | 2 | 0 | 0 | 30 | 0 | 0 | 1 | 6 | 1 | 64 |
|  |  |  | 1.4% | 0.7% | 2.1% | 2.1% | 1.4% | 22.1% | 6.2% | 2.8% | 16.6% | 16.6% | 1.4% | 1.4% | 0.0% | 0.0% | 20.7% | 0.0% | 0.0% | 0.7% | 4.1% |  |  |
| **Vancomycin** |  | 2 | 1 | 0 | 0 | 0 | 0 | 14 | 14 | 9 | 38 | 13 | 12 | 3 | 6 | 22 | 1 | 2 | 0 | 7 | 1 | 1 | 32 |
|  |  | 1.4% | 0.7% | 0.0% | 0.0% | 0.0% | 0.0% | 9.7% | 9.7% | 6.2% | 26.2% | 9.0% | 8.3% | 2.1% | 4.1% | 15.2% | 0.7% | 1.4% | 0.0% | 4.8% | 0.7% |  |  |
| **Oxytetracycline** |  |  |  |  | 5 | 8 | 6 | 1 | 9 | 9 | 6 | 6 | 8 | 12 | 16 | 51 | 2 | 4 | 2 |  |  | 16 | 32 |
|  |  |  |  |  | 3.4% | 5.5% | 4.1% | 0.7% | 6.2% | 6.2% | 4.1% | 4.1% | 5.5% | 8.3% | 11.0% | 35.2% | 1.4% | 2.8% | 1.4% |  |  |  |  |
| **Tilmicosin** |  |  |  | 1 | 0 | 1 | 0 | 6 | 7 | 8 | 24 | 8 | 5 | 4 | 8 | 14 | 45 | 5 | 2 | 1 | 6 | 32 | 64 |
|  |  |  |  | 0.7% | 0.0% | 0.7% | 0.0% | 4.1% | 4.8% | 5.5% | 16.6% | 5.5% | 3.4% | 2.8% | 5.5% | 9.7% | 31.0% | 3.4% | 1.4% | 0.7% | 4.1% |  |  |
| **Ronidazole** |  |  |  |  |  |  |  | 2 | 19 | 23 | 14 | 5 | 5 | 1 | 5 | 52 | 0 | 5 | 4 | 7 | 3 | 16 | 128 |
|  |  |  |  |  |  |  |  | 1.4% | 13.1% | 15.9% | 9.7% | 3.4% | 3.4% | 0.7% | 3.4% | 35.9% | 0.0% | 3.4% | 2.8% | 4.8% | 2.1% |  |  |
| **Metronidazole** |  |  |  | 4 | 0 | 0 | 3 | 7 | 8 | 17 | 24 | 3 | 4 | 2 | 0 | 52 | 0 | 2 | 7 | 4 | 8 | 32 | 256 |
|  |  |  |  | 2.8% | 0.0% | 0.0% | 2.1% | 4.8% | 5.5% | 11.7% | 16.6% | 2.1% | 2.8% | 1.4% | 0.0% | 35.9% | 0.0% | 1.4% | 4.8% | 2.8% | 5.5% |  |  |

*ratio 2:1
